# Severity-dependent risk of chromosomal abnormalities in fetuses with short long bones: a 10-year cohort study

**DOI:** 10.1186/s40246-026-00977-0

**Published:** 2026-05-10

**Authors:** Yanlin Huang, Hongke Ding, Jian Lu, Ting Wang, Xiaomei Shi, Cuiqing Huang, Jing Wu

**Affiliations:** 1 Medical Genetic Center, Guangdong Women and Children Hospital, Guangzhou, 511400 Guangdong China; 2 Guangzhou Key Laboratory of Prenatal Screening and Diagnosis, Guangdong Women and Children Hospital, Guangzhou, 511400 Guangdong China; 3 UItrasonic Diagnosis Deparment, Guangdong Women and Children Hospital, Guangzhou, 511400 Guangdong China

**Keywords:** Short long bones, Chromosomal abnormalities, Prenatal diagnosis, Chromosomal microarray analysis, Karyotyping

## Abstract

**Background:**

Short long bones (SLBs) detected prenatally are associated with chromosomal abnormalities; however, risk stratification according to phenotypic severity and associated anomalies remains incompletely defined.

**Methods:**

In this 10-year retrospective cohort study (2015–2024), 790 fetuses with SLBs (≥ 2 SD below the gestational mean) underwent conventional karyotyping (CS) and/or chromosomal microarray analysis (CMA). SLBs were categorized by severity (mild, moderate, severe) and presentation (isolated vs. non-isolated). Detection rates were compared across subgroups.

**Results:**

Chromosomal abnormalities were identified in 38/790 fetuses (4.8%), including 2.2% numerical and 2.7% structural aberrations. The detection rate increased with limb shortening severity (3.1% in mild, 8.2% in moderate, and 8.8% in severe cases; *P* = 0.005). Non-isolated SLBs were associated with a significantly higher abnormality rate than isolated cases (7.7% vs. 2.2%, *P* < 0.001). CMA detected clinically relevant submicroscopic copy number variants missed by karyotyping, whereas karyotyping identified balanced rearrangements not detectable by CMA.

**Conclusions:**

Chromosomal abnormalities represent a measurable but limited proportion of fetuses with SLBs, with risk significantly influenced by phenotypic severity and coexisting anomalies. These findings support a risk-stratified framework for prenatal genetic evaluation and may inform clinical counseling and management strategies.

## Introduction

Short long bones (SLBs), defined as fetal long bones measuring ≥ 2 standard deviations (SDs) below the normal range, represent a significant prenatal ultrasound finding with complex etiologies and varied clinical implications [1, 2]. Isolated SLBs often have a favorable prognosis, potentially due to constitutional, ethnic, or familial factors. However, a portion of fetuses with SLBs are associated with multiple malformation syndromes affecting various organ systems [3–6]. The incidence of chromosomal abnormalities in SLBs cases varies widely in the literature, with some studies reporting rates between 6–27% [4, 5, 7] Common chromosomal abnormalities associated with SLBs include trisomies 21, 18, and 13, as well as various microdeletion and microduplication syndromes [7, 8]. The complex etiology and non-specific phenotypic features of SLBs underscore the importance of accurate genetic diagnosis for informed parental counseling and appropriate pregnancy management.

Despite the widespread application of conventional karyotyping (CS) and chromosomal microarray analysis (CMA) in detecting chromosomal abnormalities in fetuses with structural anomalies, studies specifically addressing chromosomal aberrations in fetuses with SLBs remain scarce. The existing literature on this subject is limited, often featuring insufficient sample sizes (38–129 cases) and lacking comprehensive analysis across the full spectrum of SLBs severity [4–9]. Consequently, there is a notable lack of accurate data regarding the incidence of chromosomal abnormalities associated with SLBs, which hinders evidence-based clinical decision-making. To address this knowledge gap, we conducted a large-scale retrospective cohort study of fetuses with sonographically identified SLBs undergoing karyotyping and CMA at our institution over a 10-year period. Our study aimed to precisely evaluate the incidence and characteristics of chromosomal abnormalities in fetuses with SLBs, providing valuable insights for prenatal diagnosis and genetic counseling.

## Materials and methods

This retrospective cohort study was conducted at Guangdong Women and Children Hospital between January 2015 and December 2024. The study has been approved by our institutional review board and clinical research ethics committee. Written informed consents were obtained from all participants. All the procedures performed in the study were in accordance with the Declaration of Helsinki and as we previously described [10, 11]. 

Fetuses were included in the study if their prenatal ultrasound reports indicated the presence of SLBs. The sonographic criteria for short limb deformities include a fetal femur length (FL) measuring ≥ 2 standard deviations (SDs) below our gestational age-specific reference ranges, either in isolation or accompanied by additional abnormalities [2, 4]. The reference for diagnosing shortened long bones in a fetus is based on data from the southern Chinese population [12]. Shortened long bones were classified into three categories based on FL measurements: mild SLBs when FL was 2–3 SDs below the gestational age-specific mean, moderate SLBs when FL was 3–4 SDs below the mean, and severe SLBs when FL was ≥ 4 SDs below the mean. Isolated SLBs was defined as sole SLBs. Non-isolated SLBs was defined as SLBs in combination with another soft marker or a structural abnormality.

After diagnosis, all parents were given comprehensive counselling. Karyotyping and CMA analysis were presented to all fetuses. Metaphase chromosome G-banding karyotyping was performed at a level of 320–400 bands. CMA was performed for the proband using an Affymetrix Cytoscan 750 K GeneChip. The procedure was performed according to the manufacturer’s instruction. Data analysis was performed using the Chromosome Analysis Suite (ChAS) 4.1 software. Copy Number Variations (CNVs) larger than 100 kb or those that affected more than 50 contiguous probes were considered. The pathogenicity of candidate CNVs was evaluated consulting the Database of Genomic Variants (DGV) (http://dgv.tcag.ca/dgv/app/home), DECIPHER (https://www.deciphergenomics.org/browser), and ClinGen (http://www.ncbi.nlm.nih.gov/projects/dbvar/clingen/). The clinical significance of the candidate CNVs was evaluated according to the criteria of the ACMG and the ClinGen [13]. 

Statistical analysis was performed using R version 4.4.2 (R Core Team, 2024). Quantitative variables are expressed as the mean ± standard deviation, and categorical variables are expressed as the frequency and percentage. Chi-square tests or Fisher’s exact test were used to compare the incidence of chromosomal abnormalities, with Bonferroni correction for multiple comparisons. *P* < 0.05 was considered statistically significant.

## Results

**Fig. 1:**
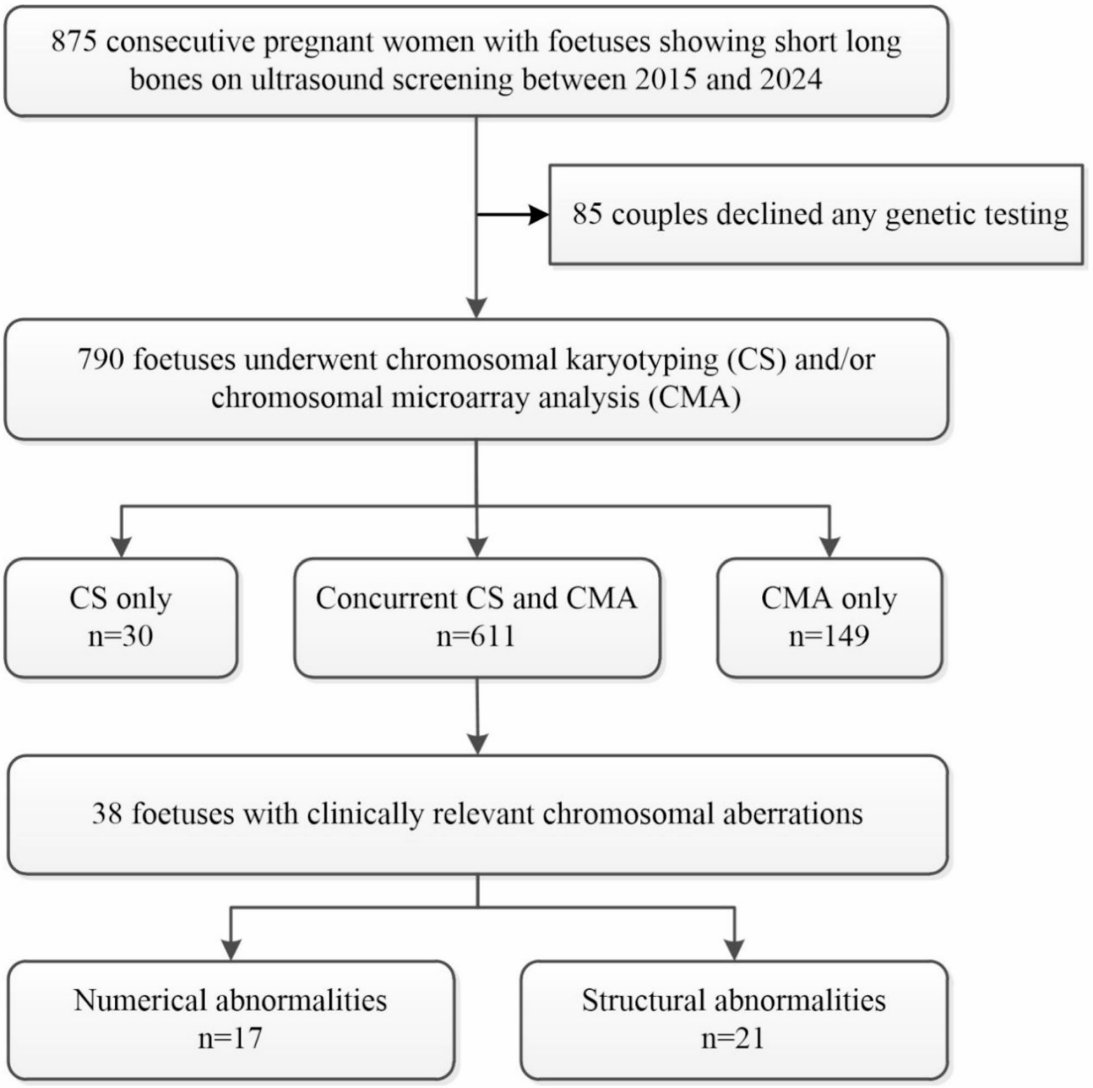
Flow chart of study enrollment and genetic testing in fetuses with short long bones

From a total of 875 consecutive pregnant women with fetuses showing short long bones on ultrasound screening between 2015 and 2024, 790 fetuses underwent genetic testing and were included in this cohort study (Fig. 1). The mean maternal age was (29.35 ± 4.58) years, and the mean gestational age was (29.33 ± 4.44) weeks. The average Z-score for SLBs severity was (-2.84 ± 1.25). SLBs were classified as mild (2–3 SDs below mean) in 68.4% (*n* = 540), moderate (3–4 SDs below) in 20.1% (*n* = 159), and severe (≥ 4 SDs below) in 11.5% (*n* = 91) of fetuses. Isolated SLBs accounted for 52.4% (*n* = 414) of fetuses, while non-isolated SLBs comprised 47.6% (*n* = 376). The primary sample types were umbilical cord blood (60.0%) and amniotic fluid (36.2%). Regarding testing methods, 49.0% of fetuses underwent both conventional CS and CMA, 18.9% had CMA only, and 3.8% had CS only (Table 1).

**Table 1:** Characteristics of the eligible subjects in this cohort

Characteristic	*n* or Mean	% or SD
Total	790	100
Maternal age (years)	29.35	4.58
Gestational age (weeks)	29.33	4.44
SLBs severity (Z-score)	− 2.84	1.25
Mild (2–3 SDs below)	540	68.4
Moderate (3-4SDs below)	159	20.1
Severe (≥ 4 SDs below)	91	11.5
SLBs category		
Isolated SLBs	414	52.4
Non-isolated SLBs	376	47.6
Sample type		
Umbilical cord blood	474	60.0
Amniotic fluid	286	36.2
Skin tissue	24	3.0
Villus	6	0.8
Test items		
Concurrent CS and CMA	611	77.3
CMA	149	18.9
CS	30	3.8

SLBs refers to short long bones; SDs refers to standard deviations; Z-score refers to how many standard deviations below the mean for fetuses of the same gestational age; CS refers to conventional karyotyping; CMA refers to chromosomal microarray analysis

### Chromosomal abnormalities detected in fetuses with SLBs

**Fig. 2:**
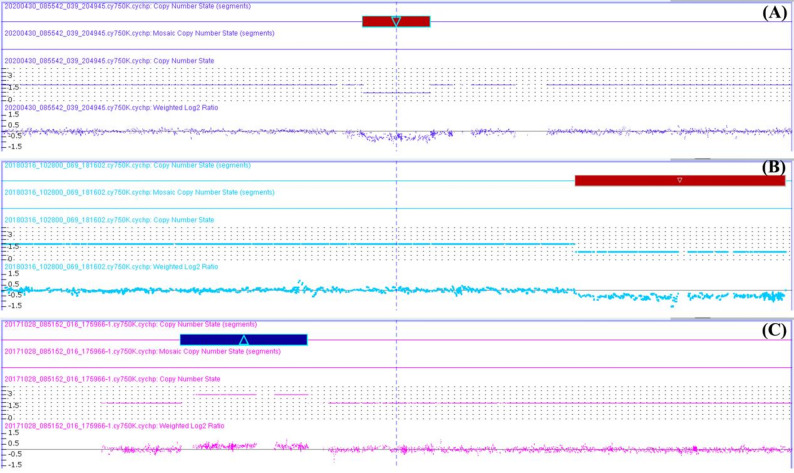
Representative chromosomal microarray analysis (CMA) results. **A** Case 1 showing a 1.5 Mb deletion at 7q11.23; **B** Case 4 showing a 1.7 Mb deletion at 22q13.33; **C** Case 15 showing a 2.8 Mb duplication at 22q11.21. The red and blue dots represent the log2 ratio of probe intensities, indicating copy number loss and gain, respectively

Chromosomal abnormalities were detected in 38 out of 790 fetuses with SLBs (4.8%) (Table 2). Of these, 17 were numerical abnormalities (2.2%) and 21 were structural abnormalities (2.7%). The most common numerical abnormality was trisomy 21 (8 cases), followed by monosomy X (3 cases). Additionally, 5 cases of aneuploid mosaicism were identified (Table 3). Among the structural abnormalities, copy number deletion-related syndromes were observed in 10 cases, copy number duplication-related abnormalities in 2 cases, and 5 cases exhibited both copy number deletions and duplications simultaneously. Moreover, one inversion and one translocation were identified by CS (Table 4). Representative CMA plots showing pathogenic CNVs (e.g., 7q11.23 deletion and 22q13.33 deletion) are presented in Fig. 2.

**Table 2:** Chromosomal abnormality incidence in SLBs of different severities and categories

Subgroup	CS positive cases (*N* = 641)		CMA positive cases (*N* = 760)		CS and/or CMA positive cases(*N* = 790)	
	*n*/*N*	%	*n*/*N*	%	*n*/*N*	%
SLBs severity (Z-score)	*P* = 0.066^*^		*P* = 0.024^*^		*P* = 0.005^*^	
Mild (2–3 SDs below)	11/416	2.64%	11/517	2.13%	17/540	3.1%
Moderate (3-4SDs below)	8/138	5.80%	10/155	6.45%	13/159	8.2%
Severe (≥ 4 SDs below)	6/87	6.90%	3/88	3.41%	8/91	8.8%
SLBs category	$\:{\chi\:}^{2}=12.084,\:$*P* = 0.001		$\:{\chi\:}^{2}=$5.225, *P* = 0.022		$\:{\chi\:}^{2}=13.202$, *P* < 0.001	
Isolated SLBs	4/321	1.25%	7/396	1.77%	9/414	2.2%
Non-isolated SLBs	21/320	6.56%	17/364	4.67%	29/376	7.7%
Total	25/641	3.90%	24/760	3.16%	38/790	4.8%

SLBs refers to short long bones; SDs refers to standard deviations; Z-score refers to how many standard deviations below the mean for fetuses of the same gestational age; CS refers to conventional karyotyping; CMA refers to chromosomal microarray analysis. ^*^refers to P-value calculated using Fisher’s exact test

**Table 3:** Chromosomal numerical abnormalities and ultrasound findings in fetuses with short long bones

No.	Z-score	Ultrasound malformations	Karyotype	Pregnancy outcome
1	−4.070	Dilation of bilateral lateral ventricles	49,XXXXY	TOP
2	−3.014	Aplasia of the nasal bone, polyhydramnios	47,XY,+21	Refused follow-up
3	−3.039	Hypoplastic heart, polyhydramnios	47,XY,+21	TOP
4	−2.544	Ventricular septal defect, echo genic fetal bowel	47,XY,+21	TOP
5	−2.552	SLBs	47,XY,+21	TOP
6	−3.226	Dilation of bilateral lateral ventricles, abnormality of the left ventricle	47,XY,+21	TOP
7	−4.164	Increased placental thickness velamentous placenta, single umbilical artery	47,XYY	Alive
8	−2.784	Widened posterior fossa	mos45,X[63]/46,XY[37]	TOP
9	−3.271	Hypoplastic heart, hydronephrosis	mos47,XY,+8[13]/46,XY [87]	TOP
10	−2.721	Thickened nuchal skinfold	45,X	TOP
11	−6.405	Ventricular septal defect	47,XY,+21	TOP
12	−4.196	SLBs	mos47,XY,+21[98]/46,XY[2]	TOP
13	−4.095	Increased fetal cardiothoracic ratio	69,XXY	TOP
14	−2.259	Dilation of left lateral ventricles, abnormality of the atrial septum, battledore placenta	mos47,XY,+7[4]/46,XY[18]	TOP
15	−4.661	SLBs	47,XX,+21	TOP
16	−2.872	Coarctation of aorta	45,X	TOP
17	−2.115	Abnormality of the atrial septum, tricuspid regurgitation	mos47,XXX[4]/46,XX[96]	TOP

SLBs refers to short long bones; Z-score refers to how many standard deviations below the mean for fetuses of the same gestational age; TOP refers to Termination of Pregnancy

**Table 4:** Chromosomal structural abnormalities and ultrasound findings in fetuses with short long bones

No.	Z-score	Ultrasound malformations	Detection results	Size(Mb)	Clinical diagnosis	Origin	Pregnancy outcome
1	−2.784	SLBs	7q11.23(72669480–74154209)x1	1.5	Williams-Beuren syndrome	NA	TOP
2	−3.712	Ventricular septal defect, persistent left superior vena cava	3p26.3p25.3(61891-11571689)x1	11.5	3p-syndrome	NA	TOP
3	−3.732	Battledore placenta	Xp22.33p11.21(168551-55476636)x1, Xp11.21q28(55754932–155233098)x3	55.3,99.5	Xp22.3 microdeletion syndrome, Xp11.21q28 duplication	NA	TOP
4	−2.438	Persistent left superior vena cava	22q13.33(49542105–51197766)x1	1.7	Phelan-McDermid syndrome	NA	TOP
5	−2.817	Dilation of left lateral ventricles, widened posterior fossa	16p13.3p13.2(85880-7964267)x3, 17q25.3(80290198–81041823)x1	7.9,0.8	16p13.3 duplication syndrome,17q25.3 deletion	NA	TOP
6	−3.806	SLBs	Xp22.33(168551–1523405)x1	1.4	Xp22.3 microdeletion syndrome	NA	TOP
7	−4.846	Cleft palate, low-lying conus medullaris	7q11.23(72701098–74133586)x3	1.4	7q11.23 duplication syndrome	NA	TOP
8	−3.032	SLBs	21q11.2q22.11(15016487–32908642)x1, Xp22.33 or Yp11.32(168552–1216144 or 118552–1166144)x1	17.9,1.1	21q11.2q22.11 deletion, Xp22.3 microdeletion syndrome	De novo	TOP
9	−2.036	Hyperechogenic kidneys, abnormality of toe, increased fetal biparietal diameter and head circumference	3q29(195740357–197310451)×1	1.6	3q29 microdeletion syndrome	NA	Alive
10	−2.287	Fetal growth restriction	16p11.2(29567296–30190029)x1	0.6	16p11.2 deletion syndrome	NA	Alive
11	−2.136	Ventricular septal defect, persistent left superior vena cava, micrognathia, polyhydramnios	18p11.32p11.21(136227-15181208)x1, 18q11.1q23(18529577–78013728)x3	15.1,59.5	18p deletion syndrome,18q11.1q23 duplication	De novo	TOP
12	−2.067	Widened posterior fossa	10q11.22q11.23(46293590–51817663)x1	5.5	10q11.22q11.23 microdeletion	De novo	Alive
13	−3.029	SLBs	6p12.3p11.2(49039846–57039030)x1	8	Char syndrome	De novo	
14	−3.561	Fetal growth restriction	16p13.11(15416497–16085105)x1	0.7	16p13.11 microdeletion syndrome	NA	Alive
15	−2.068	Ventricular septal defect, pleural effusion	22q11.21(18648855–21461017)x3	2.8	22q11.2 microduplication syndrome	NA	TOP
16	−3.316	Fetal growth restriction, oligohydramnios, single umbilical artery	16p11.2(29428531–30190029)x1	0.8	16p11.2 deletion syndrome	NA	Alive
17	−4.263	SLBs	(X)×1,Yp11.31q11.221(2650425–16088091)x1-2, Yq11.221q11.23(16174683–28799654)x0-1	13.4,12.6	Mosaic	NA	Alive
18	−2.349	SLBs	17q25.1q25.3(73720010–81041823)x3, Xp22.33p22.2(168551–9626792)x1	7.3,9.5	Xp22.3 microdeletion syndrome,17q25.1q25.3 duplication	NA	TOP
19	−2.007	Low-lying conus medullaris, sacrococcygeal dysplasia	46,XY, inv(18)(p11.2q11.2)	--	Inversion	NA	Alive
20	−3.078	Dilation of bilateral lateral ventricles, battledore placenta	Xp22.33q22.3(2709027-104760646)×3, Xq22.3q27.3(104811651–146030305)×1	104.6,41.1	Xp22.33q22.3 duplication, Xq22.3q27.3 deletion	De novo	Alive
21	−3.078	Hydronephrosis of right kidney	46,XY, t(1,7)(q43,q35)	--	Translocation	Maternal	Alive

SLBs refers to short long bones; Z-score refers to how many standard deviations below the mean for fetuses of the same gestational age; NA refers to missing data due to the test not being performed; TOP refers to Termination of Pregnancy

### Chromosomal abnormality rates increase with SLBs severity

The incidence of chromosomal abnormalities positively correlated with SLBs severity. In mild, moderate, and severe SLBs groups, chromosomal abnormality detection rates were 3.1%, 8.2%, and 8.8%, respectively (*P* = 0.005, Fisher’s exact test) (Table 2). The results for CS demonstrated an increase in abnormality detection rates from 2.64% to 6.90% with increasing SLB severity. However, this trend was not statistically significant (*P* = 0.066, Fisher’s exact test) (Table 2). CMA results demonstrated a similar trend, with detection rates rising from 2.13% to 3.41% (*P* = 0.024, Fisher’s exact test) (Table 2). Post-hoc analysis revealed significant differences between mild and moderate groups (*P* < 0.05) and between mild and severe groups (*P* < 0.05), but not between moderate and severe groups (*P* > 0.05).

### Higher chromosomal abnormality rates in non-isolated SLBs

Non-isolated SLBs exhibited a significantly higher incidence of chromosomal abnormalities compared to isolated SLBs (7.7% vs. 2.2%, $\:{\chi\:}^{2}=13.202,$ *P* < 0.001) (Table 2). CS detection revealed abnormality rates of 6.56% in non-isolated SLBs versus 1.25% in isolated SLBs ($\:{\chi\:}^{2}=12.084,\:$*P* = 0.001) (Table 2). Similarly, CMA results showed higher detection rates in non-isolated SLBs (4.67% vs. 1.77%, $\:{\chi\:}^{2}=$5.225, *P* = 0.022) (Table 2). Stratified by SLBs severity, the chromosomal abnormality rate was higher in the Non-isolated SLBs group compared to the Isolated SLBs group within the Mild and Moderate subgroups (5.88% vs. 0.99%, $\:{\chi\:}^{2}=$10.434, *P* = 0.001; 13.70% vs. 3.49%, $\:{\chi\:}^{2}=$5.483, *P* = 0.019) (Table 5). However, this trend was not observed in the Severe subgroup (7.69% vs. 11.54%, $\:{\chi\:}^{2}=$0.031, *P* = 861) (Table 5).

**Table 5:** Incidence of CS/CMA positive cases across SLBs severity subgroups

Subgroup of SLBs severity	CS and/or CMA positive cases	
	*n*/*N*	%
Mild (2–3 SDs below)	$\:{{\upchi\:}}^{2}=10.434,\:$*P* = 0.001	
Isolated SLBs	3/302	0.99%
Non-isolated SLBs	14/238	5.88%
Moderate (3-4SDs below)	$\:{{\upchi\:}}^{2}=5.483,\:$*P* = 0.019	
Isolated SLBs	3/86	3.49%
Non-isolated SLBs	10/73	13.70%
Severe (≥ 4 SDs below)	$\:{{\upchi\:}}^{2}=0.031,\:$*P* = 0.861^*^	
Isolated SLBs	3/26	11.54%
Non-isolated SLBs	5/65	7.69%

SLBs refers to short long bones; SDs refers to standard deviations; Z-score refers to how many standard deviations below the mean for fetuses of the same gestational age; CS refers to conventional karyotyping; CMA refers to chromosomal microarray analysis. ^*^refers to P-value calculated using a chi-square test with continuity correction

## Discussion

This retrospective study analyzed 790 fetuses with shortened long bones (SLBs) over a 10-year period (2015–2024), representing the largest known cohort to date. We identified a chromosomal abnormality incidence of 4.8%, including 2.2% numerical and 2.7% structural aberrations. The overall rate was lower than previous reports (6–27%), [4–7, 14] likely due to the high proportion of mild cases (~ 70%) in our cohort. Importantly, variation in reported detection rates across studies may also reflect heterogeneity in inclusion criteria, gestational age at diagnosis, population background, and testing strategies (karyotype-only versus CMA or combined testing) [4, 5, 9, 14, 15]. In cohorts enriched for severe limb shortening or additional structural anomalies, detection rates are consistently higher, suggesting that phenotypic stratification is critical when interpreting incidence data [15, 16]. 

The spectrum of chromosomal abnormalities observed in our cohort of fetuses with SLBs provides valuable insights into the genetic landscape of this condition. Among the numerical abnormalities, trisomy 21 was the most common, accounting for 47% (8/17) of aneuploidies. Mosaic aneuploidies were identified in five cases (Table 3), highlighting the critical relevance of considering mosaicism in prenatal evaluation. Notably, our combined use of CS and CMA enabled the detection of low-level mosaicism, such as the 4% mosaicism for trisomy X (Case 17) and 18% for trisomy 7 (Case 14), both of which were associated with additional ultrasound findings like atrial septal defects or ventriculomegaly. The association between trisomy 21 and shortened long bones has been well established, as limb shortening represents a recognized soft marker linked to altered skeletal growth dynamics in Down syndrome [4, 17]. In contrast, sex chromosome abnormalities, including monosomy X and mosaic variants (e.g., mos 45,X/46,XY), likely reflect disrupted *SHOX* gene dosage effects. Haploinsufficiency of the *SHOX* gene is a known driver of impaired longitudinal bone growth, providing a clear biological explanation for SLBs in these cases [18]. This biological plausibility, coupled with the potential for low-level mosaicism to manifest significant phenotypes, reinforces the importance of careful chromosomal evaluation in fetuses presenting with SLBs, even when additional anomalies are subtle.

Structural abnormalities primarily involved copy number variants (CNVs), including deletions and duplications ranging from submicroscopic to large-scale alterations. Notable recurrent regions included 7q11.23, 16p11.2, 22q11.2, and Xp22.3. These loci are known to harbor dosage-sensitive genes involved in developmental signaling pathways, including MAPK/ERK, TBX1-related cardiac–skeletal interactions, and SHOX-associated growth regulation [19–21]. Although SLBs are not the defining feature of many microdeletion/microduplication syndromes, impaired skeletal growth may represent part of a broader multisystem developmental disturbance. Therefore, SLBs may serve as an early phenotypic indicator of underlying syndromic CNVs, particularly when accompanied by cardiac, craniofacial, or central nervous system anomalies [9, 16]. Additionally, karyotyping revealed one balanced translocation and one inversion, both undetectable by CMA, underscoring the complementary value of CS and CMA. These findings support the continued clinical relevance of combined cytogenetic approaches, especially in settings where balanced rearrangements may influence reproductive counseling or recurrence risk assessment [13]. 

The incidence of chromosomal abnormalities increased with SLBs severity, from 3.1% in mild to 8.8% in severe cases, reflecting a dose-dependent relationship. This observation suggests an association between the degree of limb shortening and the likelihood of chromosomal disruption within our cohort, potentially serving as a phenotypic indicator for risk stratification. In moderate cases, the relatively higher CMA yield may reflect a mixed etiological profile, including submicroscopic CNVs contributing to partial skeletal growth restriction. The apparent plateau in chromosomal risk observed between moderate and severe groups in this study may be consistent with a transition toward monogenic etiologies in extreme phenotypes, although further prospective studies are needed to confirm this trend [6, 22–24]. This interpretation aligns with previous large prenatal sequencing studies demonstrating that the diagnostic yield of exome sequencing increases substantially in fetuses with severe skeletal phenotypes, particularly when femur length is ≥ 4 SDs below the mean [6, 22, 23]. Such cases are frequently attributable to pathogenic variants in genes regulating endochondral ossification (e.g., *FGFR3*, *COL1A1/2*, *COL2A1*, *ACAN*), which are not detectable by CMA [24–26]. 

We also observed a clear difference between isolated and non-isolated SLBs. Non-isolated SLBs showed a markedly higher incidence of chromosomal abnormalities (7.7%) compared to isolated SLBs (2.2%). This finding supports the established principle in prenatal genetics that the presence of multiple structural anomalies significantly increases the probability of chromosomal imbalance [14, 22]. Similarly, our previous exome sequencing (ES) study in this cohort revealed a substantially higher diagnostic yield in non-isolated cases (44.4%) than in isolated ones (27.3%), contributing to an overall incremental yield of 40.4% following negative chromosomal results [10]. 

In clinical practice, SLBs accompanied by cardiac defects, central nervous system anomalies, or growth restriction should prompt strong consideration of invasive testing. While CMA is a standard first-line evaluation, the integration of whole-exome sequencing (WES) with concurrent CNV analysis represents a more efficient and comprehensive approach that can significantly reduce the time required to reach a definitive diagnosis [27]. This is particularly critical given the extreme etiological heterogeneity of non-isolated SLBs, which likely reflects the coexistence of multiple pathogenic mechanisms—including chromosomal aneuploidy, pathogenic CNVs, and single-gene skeletal dysplasias. Therefore, while a stepwise testing strategy is often employed—(1) karyotyping and CMA; (2) reflex exome sequencing—early consideration of WES with integrated CNV analysis may optimize diagnostic turnaround time and improve clinical decision-making [22, 23, 28]. 

CMA and CS each demonstrated complementary diagnostic value. CMA identified clinically relevant microdeletions missed by CS (~ 0.8%), whereas CS detected balanced rearrangements inaccessible to CMA. Although the incremental yield of CMA over karyotyping appears modest in absolute percentage terms, it represents clinically actionable diagnoses with important prognostic and recurrence implications [13, 19]. Furthermore, the detection of pathogenic CNVs may influence perinatal management decisions and long-term developmental surveillance. Nonetheless, both CS and CMA are limited in detecting single-gene disorders. Our prior WES study in this cohort revealed pathogenic variants in 40.4% of tested cases, with skeletal dysplasias comprising 60.7% of diagnoses [10]. Most notably, the diagnostic yield in fetuses with severe limb shortening (FL < -4 SDs) reached 72.5%, which was significantly higher than the 16.7% observed in cases with milder shortening [10]. When contextualized within the broader prenatal genomics literature, these findings are consistent with PAGE and related cohort studies demonstrating an incremental diagnostic yield of approximately 8–15% for exome sequencing in unselected structural anomalies, but substantially higher yields (30–60%) in selected skeletal phenotypes [22–24, 28]. Therefore, severe SLBs—particularly when FL is ≥ 4 SDs below the mean—should be considered a high-priority indication for prenatal exome sequencing, provided appropriate pretest counseling is performed regarding secondary findings and variants of uncertain significance [22, 23, 28]. 

From a developmental biology perspective, fetal long bone growth depends on coordinated chondrocyte proliferation, hypertrophy, extracellular matrix deposition, and vascular invasion within the growth plate. Disruption of these pathways may arise from chromosomal dosage imbalance, single-gene defects, or placental insufficiency [7, 24]. In mild isolated SLBs, constitutional or ethnic variation and placental-mediated growth restriction may predominate [7, 8]. In contrast, severe early-onset shortening is more frequently associated with intrinsic skeletal dysplasia or chromosomal pathology [6, 24, 26]. This phenotypic continuum underscores that SLBs represent not a single diagnostic entity but a heterogeneous ultrasonographic sign requiring etiologically stratified evaluation.

Clinically, our data support a risk-stratified counseling framework:

- Mild isolated SLBs: These cases carry a relatively low chromosomal abnormality risk (1–3%). Individualized counseling should consider parental stature and serial growth assessment. In such cases, cffDNA-based screening (NIPT) may be considered a useful non-invasive option to refine the risk of common aneuploidies, while invasive testing remains available based on clinical evolution or patient preference.
- Moderate SLBs or non-isolated cases: These present an intermediate risk (8–14%); invasive testing with CMA is recommended as the first-line evaluation.
- Severe SLBs (≥ 4 SDs): These carry a high suspicion for monogenic skeletal dysplasia (with a diagnostic yield of 72.5% for WES); early integration of exome sequencing should be considered to optimize diagnostic turnaround time.

Such stratification may improve cost-effectiveness and reduce unnecessary invasive procedures while maximizing diagnostic yield [28, 29]. 

This study has several limitations. First, its retrospective design may introduce referral bias, particularly toward more severe phenotypes in a tertiary center. Second, not all cytogenetically negative cases underwent exome sequencing, likely underestimating the true genetic contribution to SLBs. Furthermore, while a significant trend was observed across severity groups, the lack of statistical difference in chromosomal risk between the moderate and severe subgroups (*P* > 0.05) may be limited by the sample size of these specific strata; thus, these results should be interpreted with caution. Third, long-term postnatal follow-up was incomplete in continuing pregnancies, limiting genotype–phenotype correlation. Future prospective multicenter studies integrating standardized imaging protocols, genomic testing, and longitudinal follow-up are warranted.

## Conclusions

In conclusion, this large-scale cohort demonstrates that chromosomal abnormalities account for a measurable but limited proportion (4.8%) of fetuses with short long bones, with significantly higher rates observed in non-isolated and moderate-to-severe cases. The risk gradient according to limb shortening severity underscores the value of phenotypic stratification in prenatal risk assessment. While combined CS and CMA provided complementary diagnostic insights in this cohort, the relatively modest chromosomal detection rate in severe cases is consistent with the possibility that monogenic skeletal disorders represent a substantial underlying etiology in this subgroup. These findings may serve as a reference for risk-adapted genetic counseling and individualized pregnancy management, while acknowledging the need for multi-center validation.

## Data Availability

The data that support the findings of this study are available from the corresponding author upon reasonable request.

## Abbreviations

SLBs: Short long bones
SDs: Standard deviations
CS: Chromosomal karyotyping
CMA: Chromosomal microarray analysis
FL: Femur length
CNVs: Copy number variations
